# The Hi Five study: design of a school-based randomized trial to reduce infections and improve hygiene and well-being among 6–15 year olds in Denmark

**DOI:** 10.1186/s12889-015-1556-1

**Published:** 2015-03-01

**Authors:** Anette Johansen, Anne Maj Denbæk, Camilla Thørring Bonnesen, Pernille Due

**Affiliations:** Centre for Intervention Research in Health Promotion and Disease Prevention, National Institute of Public Health, University of Southern Denmark, Øster Farimagsgade 5A 2., København K, DK-1353 Denmark

**Keywords:** School, Child, Intervention, Hand wash, Infectious illness, Illness-related absenteeism, Randomized trial, Design, Effect evaluation, Process evaluation

## Abstract

**Background:**

Infectious illnesses such as influenza and diarrhea are leading causes of absenteeism among Danish school children. Interventions in school settings addressing hand hygiene have shown to reduce the number of infectious illnesses. However, most of these studies include small populations and almost none of them are conducted as randomized controlled trials. The overall aim of the Hi Five study was to develop, implement and evaluate a multi-component school-based intervention to improve hand hygiene and well-being and to reduce the prevalence of infections among school children in intervention schools by 20% compared to control schools. This paper describes the development and the evaluation design of Hi Five.

**Methods/design:**

The Hi Five study was designed as a tree-armed cluster-randomized controlled trial. A national random sample of schools (n = 44) was randomized to one of two intervention groups (n = 29) or to a control group with no intervention (n = 15). A total of 8,438 six to fifteen-year-old school children were enrolled in the study. The Hi Five intervention consisted of three components: 1) a curriculum component 2) mandatory daily hand washing before lunch 3) extra cleaning of school toilets during the school day. Baseline data was collected from December 2011 to April 2012. The intervention period was August 2012 to June 2013. The follow-up data was collected from December 2012 to April 2013.

**Discussion:**

The Hi Five study fills a gap in international research. This large randomized multi-component school-based hand hygiene intervention is the first to include education on healthy and appropriate toilet behavior as part of the curriculum. No previous studies have involved supplementary cleaning at the school toilets as an intervention component. The study will have the added value of providing new knowledge about usability of short message service (SMS, text message) for collecting data on infectious illness and absenteeism in large study populations.

**Trial registration:**

Current Controlled Trials ISRCTN19287682, 21 December 2012.

## Background

Infectious illnesses such as influenza or diarrhea are leading causes of illness and absenteeism among school children [1-5]. Illnesses among school children have a number of side-effects in addition to the direct effects on the child’s wellbeing. Illness-related school absenteeism may lead to negative educational outcomes [6,7]. Other negative consequences of illness among children are that working parents may need to take time off from work to take care of their ill child, transmission from an infected child to especially family members [8], but also to school peers or teachers, and public cost for physician visits, physician-prescribed antibiotics, and hospitalizations [9].Therefore, in addition to the above mentioned health related and educational outcomes for the child itself, childhood infectious illnesses may have a significant economic and social impact on the community.

Hands are the primary mode of transmission of many infectious illnesses particularly among school children [10]. Proper hand hygiene is generally accepted as the best means to prevent the transmission of infections. Hand washing with soap has been cited by the World Health Organization (WHO) as the most important hygiene measure in preventing the spread of infections [11,12].

Educational interventions to promote hand washing in school settings seem to improve knowledge and awareness about appropriate hand hygiene, and increase adherence with hand washing [10,13-15].

Studies of hand hygiene interventions in school settings in developed countries have shown an overall reduction in the number of infectious illnesses [16-27] although one large randomized study showed no effect [3]. Through hand hygiene interventions in the form of hand washing, use of hand disinfections and often supplemented with educational efforts, school-based interventions have reduced illness-related absenteeism among school children.

In Denmark, toilet facilities at schools have been discussed frequently by the press, and by schools, children and parents. School toilets are often run-down, dirty and lack basic amenities such as paper and soap [28,29]. Functional toilet and hand washing facilities for children are important to minimize the incidence of infectious illnesses. If the facilities provided are inadequate or uninviting, proper hand washing is less likely to take place [30]. Many school children report that they try to avoid using the school toilets or do not use them at all [28,29,31]. Avoidance of school toilets may have serious negative consequences for children, such as higher risk of incontinence, constipation and/or urinary tract infections [32]. Furthermore, school toilets, may be the only location with access to sinks, water, soap and paper. Therefore, if the children are disinclined to use the school toilets, they will probably not wash their hands during a school day.

Given the rationale above, the aim of the Hi Five study was to develop, implement and evaluate a sustainable and easily applicable multi-component school-based intervention to improve hand hygiene and school well-being and reduce the prevalence of infections by 20 percent among children at intervention schools compared to control schools.

The aim of this paper is: 1) to describe the components of the Hi Five intervention and how it was developed 2) to describe the design, planning and content of the process and effect evaluation of the program and 3) to examine whether the randomization into intervention and control schools resulted in comparable groups.

## Methods/design

### The Theoretical model

**Figure 1:**
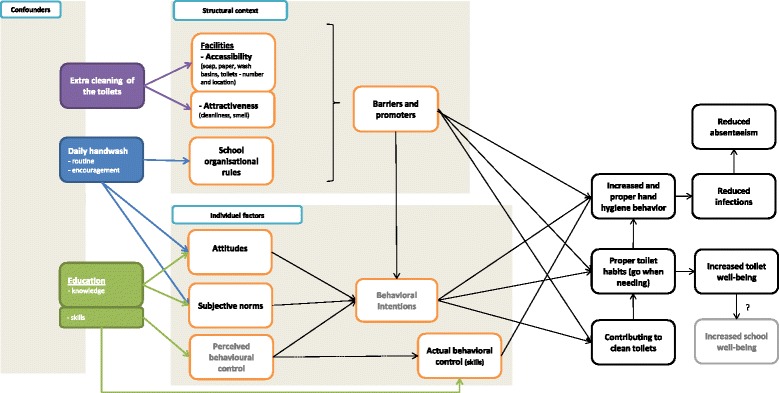
**The conceptual model of the Hi five study.**

Inspired by the Theory of Triadic Influence and a thorough review of the literature about hand hygiene interventions in school settings, we developed the conceptual model for the Hi Five study. The conceptual model includes a structural aspect, inspired by the use of the theoretical model in the “Pro Children project” [33] (Figure 1). This structural aspect includes barriers and promoters for school children’s intentions and opportunities at school for correct hand hygiene e.g. availability of soap, amount of washbasins and smell at the toilets. At the individual level the school children’s intentions to wash hands are assumed to be predicted by three intermediate variables: attitudes, subjective norms and perceived behavioral control. However, perceived control may only predict behavior to the extent that it reflects actual control. Actual behavioral control refers to the extent to which the resources and skills are available for the person to perform the desired behavior [34]. In our understanding, intentions are perceived to be less conscious among children and we assume that structural factors often influence a child’s intentions and choices. In other words the structural factors determine what choices are realistic and most likely to be operationalized and made routine [14,35].

The Intervention Mapping protocol was used to inspire the development of the study [36].

### Development of the intervention

We reviewed existing studies of school-based hygiene interventions in developed countries. Almost all of the existing interventions were multicomponent with a curriculum component supplied with extra hand washing [18,20,22] or use of hand disinfection [17,21] or consisted of single component interventions with use of hand disinfection [19,24,25]. Interventions comparing hand washing to hand disinfection showed no significant differences in reducing children’s illness-related absenteeism [37]. We deliberately choose hand wash over hand sanitizers because it is a more sustainable intervention to teach children to wash their hands properly and to encourage regular hand washing. Soap and water are the amenities available to children in most settings of their daily life, including the home.

We did not find any studies examining the association between school toilet/wash basin facilities and illness rates nor did we find any intervention aimed at improving hygiene at school toilets or children’s inclination to use the school toilets. A study performed on one elementary school in Ohio combined a hand hygiene component with a classroom surface disinfection component. This study succeeded in reducing the absenteeism rate for gastrointestinal illness in the intervention group compared with the control group [25].

Based on the above knowledge, the Hi Five study was designed to include three main intervention components: 1) a curriculum component addressing knowledge and skills 2) daily hand washing before lunch 3) extra cleaning of school toilets during the school day. Each component is described below.

#### Design

The Hi Five study is a tree-armed cluster randomized controlled trial (RCT). As children transmit illness among one another, the randomization was performed at the school level, to be able to adjust for the clustered nature of infectious illnesses (Intra-Cluster Correlation (ICC)). The schools were randomized into one of two intervention groups or to a control group with no intervention. Effectiveness of the Hi Five intervention will be evaluated using a baseline measurement and a 12 month follow-up. The effect of the intervention will be tested as an intention to treat-analysis using multilevel mixed effects models. Later analyses will explore potential differentiated effects by covariates (gender, grade, family socio economic position (SEP)).

#### Power calculations

We wanted to be able to detect a 20% reduction in the number of days the children were absent from school due to infectious illness. The sample size was based on the clustered nature of the design (school level), and an ICC of 0.10. To assess the adequate sample size of schools and children to reduce the number of days absent due to infectious illness by 20% among children in intervention schools compared to control schools, we calculated the power of a two-sample t-test using the SAS computer code suggested by Donner & Klar, 1996 [38]. The analyses showed that, with an ICC of 0.10, a rate of four absente days per child and a power of 80%, we needed 11 schools in each arm.

#### Setting

Denmark has 98 municipalities. The Danish public school consists of year 0 (preschool class) and year 1 to 9. All 10 years are mandatory. The Danish children start school the year they turn 6 [39]. Children who start together in the same class at year 0 will often belong to the same class/group of children throughout at least the first 7 years if not all ten years of schooling. There is a limit of 28 children per class. Schools have 1–4 parallel classes. There is no grouping by ability in the Danish schools e.g. all children have joint lecturing. Eighty-five percent of all Danish children attend public schools and these schools are area-based [40].

#### Population

**Figure 2:**
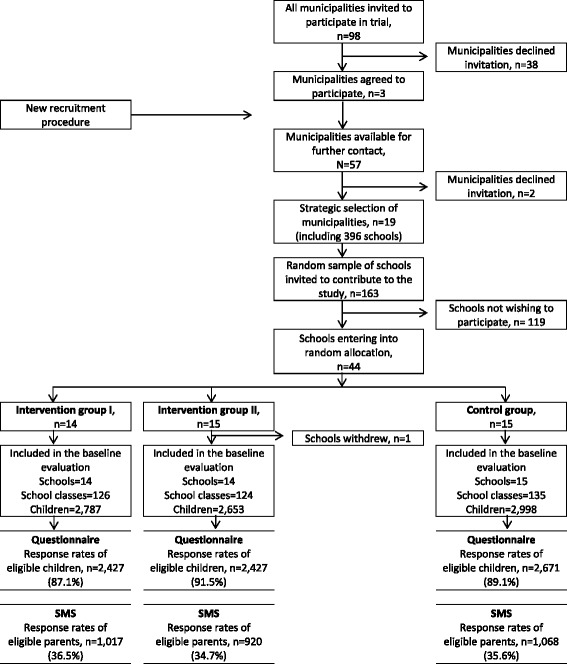
**Flow diagram of sampling, recruitment, randomization and participation of municipalities, schools and children.**

All 98 municipalities in Denmark were invited to participate in the study. Municipal staff in charge of health promotion and schools received an invitation with information about the study by mail. Municipalities were asked to finance extra cleaning of school toilets for schools randomized to receive an intervention including extra cleaning (account for one third of participating schools). The following weeks, the municipalities were contacted by telephone to follow-up on the invitation to take part in the study. Only three municipalities agreed to finance extra cleaning of school toilets. Thirty-eight municipalities declined to take part, and 57 never responded to the invitation or the follow up phone call. As a result of the low commitment rate, we decided to contact the schools directly. Consequently, we decided to apply for finance for the extra cleaning beyond the municipalities. We informed the remaining 57 municipalities about the new recruitment procedure by mail. Two municipalities denied contact to their schools. Among the remaining municipalities, 19 municipalities were strategically selected to ensure geographical spread. Together with the three municipalities who had already agreed to participate, we ended up with 22 municipalities. In these municipalities the names of all public schools were placed in an envelope, drawn randomly and listed in numbered order. The school principals at the schools in each municipality were contacted by telephone, introduced to the project and asked if we were allowed to send information material to the school. The following weeks, the schools were contacted again to ensure the material had reached them and to ask whether they wanted to participate in the study. 163 schools were contacted and 44 schools agreed to participate in the study (Figure 2). Exclusion criteria were special schools and schools planning to merge with other schools in the study period.

#### Randomization

The 44 recruited schools were randomly allocated into intervention arm I or arm II or to a control group by simple drawing. 14 schools were randomized to intervention I (curriculum component and daily hand washing before lunch), 15 schools to intervention II (curriculum component, daily hand washing before lunch and extra cleaning of school toilets) and 15 schools to the control group (no intervention). The control schools were encouraged to continue as originally planned before being contacted by the project and not to initiate any new hygiene initiatives besides those planned already. After randomization, one school in intervention group II withdrew from the study leaving 14 schools in intervention arm II. At each school, data was collected from one school class at each grade level (0- 8^th^).

### Intervention components

#### The curriculum component

All classes (n = 672) at the 28 intervention schools received the curriculum component consisting of five lessons in grade 0-3^th^ and six lessons in grade 4-9^th^ during the fall 2012. The educational material included: 1) an introduction to micro-organisms, 2) information on good practices to avoid transmission of infections, 3) instruction for practical exercises on how to wash hands consistent with the Danish Health and Medicine Authority recommendations [41], 4) information on why it is important to use the toilet and 5) material on school children’s co-responsibility to keep the school toilets clean. The educational material was tailored specifically for three age groups: 0-3^th^ grade, 4-6^th^ grade, 7-9^th^ grade (Table 1 provides further details).

**Table 1:** **Content of the Hi Five curriculum component**

**Subject**	**Intervention**	**Goal**
**0-3** ^**th**^ **grade (age 5–9)**		
**Introduction to microbes**	•Lesson in bacteria and microbes	Knowledge:
		•know that microbes are found everywhere
		•know that some microbes can help us stay healthy while others make us ill
**Spread of infection/hand hygiene**	•Discussion on how you can prevent the spread of infections	Knowledge:
	•Learning when you have to wash hands	•know that infections may be spread via hands or objects e.g. door knobs
		•know that hand washing can prevent the spread of infections
		•know in what situations and when hand wash is necessary
		•know that covering the mouth with a tissue when sneezing or coughing can prevent the spread of infections
		•know that it is more hygienic to sneeze in the sleeve than in the hands
**Step-by-step hand wash technique/practice**	•The teacher shows the children how to wash hands properly by use of an instruction CD and/or an YouTube movie.	Knowledge:
	•The children try to wash hands correctly in small groups Oil with glimmer is used to demonstrate the importance of using soap and thorough washing	•know how to wash hands correctly
	•Certificates and stickers are distributed to each child after correct hand wash	Skills:
		•be able to wash hands correctly
**Use of school toilets**	•Dialogue about why it is important to use the toilet	Knowledge:
	•Dialogue about the children’s use and experiences of the school toilets	•know that it is important with regular toilet use
	•Dialog about what the children can do so that the toilets appear more inviting	•know that they have a co-responsibility for the cleaning standard of the school toilets
	•Preparation of toilet rules	Skills:
		•be able to keep toilets more inviting
**4-6** ^**th**^ **grade (age 10–12) and 7-9** ^**th**^ **grade (age 13–15)**		
**Introduction to microbes**	•Lesson in the different types, shapes and sizes of microbes (bacteria, viruses and fungi), and where microbes are found	Knowledge:
		•know that there are three types of microbes: Bacteria, viruses and fungi
		•know that microbes are found everywhere
		•know that some microbes can help us stay healthy while others make us ill
**Spread of infection/hand hygiene**	•Through a classroom experiment children learn how microbes can spread from one person to another through touching and why it is important to wash hands properly. Small YouTube movies e.g. ‘A sneeze in slow-motion’ or ‘How long can a sneeze spread’ may be showed	Knowledge:
		•know that infections can be spread via hands or objects e.g. door knobs
		•know that hand washing can prevent the spread of infections
		•know in what situations and when hand wash is necessary
		•know that covering the mouth with a tissue when sneezing or coughing can prevent the spread of infections
		•know that it is more hygienic to sneeze in the sleeve than in the hands
**Step-by-step hand wash technique/practice**	•The teacher shows the children how to wash hands properly by use of an instruction CD or an YouTube movie	Knowledge:
	•The children try to wash hands correctly in small groups. Ultra-violet light-sensitive cream is used to demonstrate the importance of using soap and thorough washing	•know how to wash hands correctly
**Use of school toilets**	•Dialogue about why it is important to use the toilet and the children’s use of the school toilets	Skills:
	•Discussion and observation of the standard of the school toilets	•be able to wash hands correctly
	•Dialog about what the children can do so that the school toilets appear more inviting	Knowledge:
		•know that it is important with regular toilet use
		•know that they have a co-responsibility for the cleaning standard of the school toilets
		Skills:
		be able to keep toilets more inviting

*The curriculum component* for the 0-3^th^ graders consisted of the already existing material “Learn to wash your hands properly” [22] supplied with extra material on why it is important for the children to use the toilet and their co-responsibility for the cleaning standard of the school toilettes. The supplementary educational material was developed by the project group on the basis of key topics identified in the literature and with inspiration from the material ‘Clean day - good day’ [42]. An earlier draft of the educational material was discussed with two teachers from some of the intervention schools and revised accordingly.

*The curriculum component* for the 4-6^th^ graders and the 7-9^th^ graders consisted of selected lessons (1.1, 1.2, 1.3, 2.1, 2.2) and exercises from the E-bug material [43] supplemented with relevant YouTube movies and material on why it is important for children to use the school toilets and their co-responsibility for the cleaning standards for the school toilettes (based on the material ‘Clean day - good day’). The quality of the material and exercises, and the time scheduled was discussed with two teachers and an infection control nurse and revised accordingly.

As part of the material on school children’s co-responsibility to keep the school toilets clean, all intervention schools were provided with stickers designed to be mounted inside the toilet bowls, to inspire male children to aim carefully when they urinate and help them hit inside the toilet bowl.

#### The hand hygiene component

The school children were required to wash hands before lunch and the teachers were required to build in time for hand washing and to remind the children to wash their hands every day. The schools had to ensure that soap and paper towels or other drying or blowing equipment to dry hands were available at all washbasins. To support this component, schools were given stickers with step-by-step hand wash instructions to be posted next to the washbasins. School classes with 0-3^th^-graders were also provided a calendar and stickers, for keeping score with the daily hand washing. Each class was given a sticker to place on the calendar every day when the class had washed their hands.

#### Extra cleaning of school toilets

Schools in intervention arm II received both the curriculum component and the hand hygiene component, but also extra cleaning of school toilets during the intervention period. The extra cleaning was carried out by the cleaning companies already affiliated to the schools. A standard contract was developed in collaboration with a cleaning consultant, describing intervention component: Extra cleaning was carried out between 10.30 am and 12.30 pm including cleaning of toilets and sinks, removing of visible dirt, emptying bins, and refilling soap, toilet paper and paper towels.

### Implementation

Each school selected a school coordinator for the study after they had accepted to participate in the project. The school coordinators task was to receive and redistribute information concerning the Hi Five intervention and evaluation to teachers, school children and parents and to work as a Hi Five ambassador. All schools were visited by Hi Five-staff before the project was started and once again at the beginning of the school year in which the intervention was planned to be implemented. At the first meeting, the intervention was introduced, and the schools were informed about the expectations in accordance with the Hi Five intervention and the process and effect evaluation of the study. The school coordinator, the school principal, the technical administrative manager, the school nurse and a teacher representative from each departments/team were invited to be present at the second meeting when intervention elements were introduced.

The *curriculum component* was expected to be implemented in the period between September and October 2012. The hand washing component was expected to be implemented in continuation hereof and to continue throughout of the school year 20012/2013. Extra cleaning of school toilets was implemented from August 2012 to June 2013.

### Data collection

We collected the following data for the study: 1) Weekly registration of school children’s illness-related absenteeism and specification on illness, plus information on parents need to stay home from work to take care of the child, reported by the parents by mobile phone Short Message Service (SMS, text messaging) 2) Weekly registration of school teacher’s illness-related absenteeism and specification on illness, reported by the teachers themselves by SMS 3) Self-reported questionnaires from children 4) Bacteria samples from children’s hands and from school toilet facilities and 5) Observations and pictures of school toilet facilities.

#### Parents registration of their children’s illness and infection

Parents were recruited via their child’s school to participate in a 22-week illness and absenteeism registration-study, using mobile phone short message service (SMS, text messaging) for weekly data collection.

Only parents of children in the evaluation classes were invited to participate in the SMS-study. Parents enrolled in the SMS-study by returning an enrollment form distributed to the children at the schools or by filling out an electronic form available from the school intranet. When parents signed up for the SMS-study, they received an email with a detailed definition on illness categorization including two posters to print: one for the refrigerator and a small one to keep in the purse.

Each Sunday afternoon, parents received a SMS with the following question: *‘How many days has (the name of the child) been home due to illness in week xx (the week number)? Write 0 if no days, write 34 if he/she has been ill Wednesday and Thursday (0. None, 1. Monday, 2. Tuesday, 3. Wednesday, 4. Thursday, 5. Friday, 6. Saturday, 7. Sunday*)’. If the child had been ill, two further questions were sent automatically: A) ‘*What was the cause of [his/hers] illness? 1. Cold, sore throat, ear-ache, sinus infections, influenza. 2. Diarrhea, vomiting. 3. Impetigo abscesses. 4. Other infectious illness such as fever of unknown cause, chickenpox, cystitis. 5. Other illnesses such as headache, injuries, asthma* and B) ‘*Did the child’s mother or father have to stay home from work to care for the ill child? 1. No, we found another solution 2. No, he/she was home alone 3. Yes, one day 4. Yes, two days 5. Yes, three days 6. Yes, four days 7. Yes, five days.* If the parents did not answer the question within two days, a reminder was automatically sent.

The SMS-baseline data collection was conducted from the beginning of December 2011 to the end of April 2012. The follow-up data collection was conducted a year later. To motivate parents to sign up for the study and to keep response rates high, we drew lots for a small prize by the end of each month and for a larger prize by the end of the data collection.

The SMS-questions were developed based on previous Danish studies including measures of children’s acute illnesses [1,2,44]. Questions and response categories were revised in consultation with practitioners and health visitors. To test the SMS-data collection technique, the phrasing of the SMS’ and the guidance for answering the SMS-question, a small pilot study was conducted over five weeks in July 2011, including 24 parents. The study was subsequently slightly revised.

#### Teachers registrations of illness and infection

The school staff was recruited to answer questions about their own illness on a weekly basis, similar to the data collection on children’s illness among parents. The recruitment procedure and data collection was conducted in the same way as described above. Each Sunday afternoon, the school staff received a SMS with the following question: *‘How many days have you been home due to illness in week XX (the week number)? Write 0 if no days, write 34 if you have been ill Wednesday and Thursday (0. None, 1. Monday, 2. Tuesday, 3. Wednesday, 4. Thursday, 5. Friday, 6. Saturday, 7. Sunday)*. If the teacher had had any absenteeism due to illness, a following question was sent: *What was the cause of the illness? 1. Cold, sore throat, ear-ache, sinus infections, influenza. 2. Diarrhea, vomiting. 3. Impetigo abscesses. 4. Other infectious illnesses such as fever of unknown cause, cystitis. 5. Mental illness such as stress or depression. 6. Other illnesses such as headache, injuries, asthma.* Due to a four-week lockout of teachers from Danish schools during the follow-up period, it was decided to stop the SMS-data collection among school staff at the beginning of April 2013, 3 weeks ahead of schedule, as teachers declined to respond during the conflict situation.

#### Self-reported questionnaires from children

Information on determinants, proximal outcomes and supplementary outcome measures was gathered through self-administrated internet-based questionnaires. The questionnaires were developed in two versions as described below; one applicable for 0-4^th^ grade children and another for 5-8^th^-grade children.

Children answered the internet-based questionnaires in the classroom or in a computer room after a standardized instruction given by a teacher or by Hi Five staff. The children were informed that participation was voluntary and that their answers would be treated confidentially. Teachers were asked to encourage absentees to answer the questionnaires later, either at home or at school.

The baseline data collection among children was planned to be conducted from February 2012 to April 2012. Follow-up data were planned to be collected from February 2013 to April 2013.

#### Self-reported questionnaire for 0-4^th^ grade

Before development of the electronic questionnaire for the youngest children, we conducted a systematic literature search of the validity of questionnaires for children in the age group from 5 to 10 years. Children in this age group are unable to answer questions on behavior or information of others, e.g. questions about mother and fathers level of education [45]. Based on the literature we decided to limit timespans used in questions, so that young children would only be asked to think a week back in time, and we limited the number of response categories to a maximum of three [46]. Due to the limited amount of existing validated questionnaires aimed at this age group, the questions were mainly developed by the project group. Whenever possible, questions were inspired by two Danish questionnaires for children in this age group developed by the National Council for Children and the Danish Centre of Educational Environment, respectively. Due to the limited reading skills of this age group, a ‘speaking questionnaire’ was developed. The questions and response categories were read aloud to the children by clicking on the text shown on the screen of the computer. Design and use of a speaking questionnaire was inspired by the same two Danish questionnaires. The questionnaire was designed so that it was possible to log-in to a school computer, and log-in to the internet-based questionnaire and fill it out within one lecture of 45 minutes. The baseline questionnaire was pilot tested on 24 children (14 in 0^th^ grade, six in 1^th^ grade, two in 2^nd^ grade and four in 4^th^ grade) and revised according to observations and the children’s comments. All schools were offered assistance by two Hi Five employees when the youngest children had to answer the questionnaire, to ensure that there were enough adults to help them get started, to take care of technical problems, and to help the children understand the questions, if problems occurred.

#### Self-reported questionnaire for 5- 8^th^ grade

Socio-demographic questions and questions about the children’s wellbeing were selected from validated questions from the Health Behaviour in School-aged Children (HBSC)-study [47]. The questions addressing children’s symptoms were also inspired by the HBCS-study, but we chose shorter time intervals to ensure that we would able to compare questions from this age group with questions from the youngest children. The questions on illness and toilet facilities and the school children’s use of toilets were inspired by two Danish reports about children perspective on and use of school toilets [28,48]. Questions specifically related to children’s hand hygiene and illnesses were developed by the project group. The baseline questionnaire was first pilot tested in 5^th^ grade (15 boys and 15 girls) and revised according to observations and the children’s comments. Afterwards, the questionnaire was pilot tested again. The last pilot test resulted in very minor adjustments.

#### Observations of toilet facilities and cleaning standards for school toilets

We did not have the resources to inspect all toilets at all schools, so we randomly selected two toilets at each school. The one used by children in 2^th^ grade and another used by children in 6^th^ grade. The same toilets were observed at baseline and follow-up. The observations were carried out after the 10 o’clock break for toilets used by the 2^th^ grade children and immediately after the lunch break at toilets used by the 6^th^ graders. In a standardized observation scheme, the number of toilet blocks, number of washbasin, access to hot water, hand hygiene products, and drying facilities, cleanness, access to toilet paper, feeling of privacy etc. was registered. At the same time, photos of the toilets were taken. The observation scheme was developed based on existing knowledge about 1) what environmental aspects are the most critical for bacterial transmission, 2) factors influencing hand washing behaviors and 3) factors influencing children’s use of toilets [28,29].

#### Bacteria samples

Bacteria samples were taken from the observed toilets both at baseline and follow-up. At each toilet, samples were taken from the following five places: toilet seats, inside doorknob, floor (in front of toilet), sink and one optional critical place selected by the researcher. Tryptic Soy Agar (TSA) contact plates with Neutralizer were used. The aim of this part of the study was to assess the hygienic conditions at the schools toilets.

#### Micro flora on hands

Micro flora samples were taken from nine randomly selected children visiting each of the observed toilets at baseline (n = 774) and follow-up (n = 774). Children were asked to participate after using one of the toilets. The child was requested to put one hand into a bag with 100 mL sterile water. The bag was then occluded around the wrist, and the hand was shaken in a standardized manner by an investigator for 30 seconds [49]. The samples were examined for total number of bacteria, presence of Staphylococcus aureus and presence of faecal flora.

#### Pictures of school toilets

Two students (one girl and one boy) from each of the 14 intervention schools with extra cleaning were recruited to use the mobile phones to take pictures of the school toilets and send them to the project group using Multimedia Message Service (MMS). The children were recruited directly by the researcher at the schools or through their parents. The purpose of this study was to document and observe the cleaning standards at the school toilets, and to give the children a possibility to show the toilets from their view. The participating children received a SMS every day at 10:30 am with information on which toilet they had to photograph that day (day 1: the toilet used by 1^th^ graders, day 2: The toilet used by 7^th^ graders etc.). The children were asked to take two pictures every time - one showing the whole toilet room and one showing something the children thought was important for us to see. The baseline data collection of toilet pictures was conducted in March 2012. The follow-up data collection was conducted in May 2013, two months delayed due to the lockout of the Danish teachers.

### Effect evaluation

The primary outcome measures for the effect evaluation are 1) the frequency of absence episodes and the number of days absent due to infectious illness among children measured over five months at baseline and follow-up 2) the frequency of absence from school due to illness measured by children’s self-reported questionnaires. Secondary outcome measures include children’s self-reported hand washing behavior (before lunch, after toilet visits, use of soap etc.), use of the school toilets and well-being, parents’ absence from work due to the children’s infectious illness (SMS-registration), frequency of teachers’ absence episodes and the number of days off due to infectious illness measured over five months at baseline and follow-up (from SMS-registration by teachers of own illness and infection). Proximal outcome measures include norms of and attitudes towards hand washing, knowledge of bacteria and spread of infectious illness, toilet facilities and cleaning standard of the schools toilets (bacteria samples, observations and photographs of the school toilets) (Table 2).

**Table 2:** **Outcome measures at baseline and follow-up in the Hi Five study**

**Outcome**	**Collected by**	**Timing of collection**	**Source**
**Primary outcomes**			
Number of absence episodes due to infectious illness	SMS	Every Sunday afternoon December to April	Parents
Number of days absent due to infectious illness	SMS	Every Sunday afternoon December to April	Parents
Frequency of illness-related absenteeism from school last week	Internet-based questionnaire	One day between February and April	Children
**Proximale outcomes**			
Norms of and attitudes towards hand washing	Internet-based questionnaire	One day between February and April	Children
Knowledge about bacteria and spread of infectious illness	Internet-based questionnaire	One day between February and April	Children
**Secondary outcomes**			
Hand washing behavior	Internet-based questionnaire	One day between February and April	Children
Use of school toilets	Internet-based questionnaire	One day between February and April	Children
Well-being	Internet-based questionnaire	One day between February and April	Children
Parents’ absence from work due to their child’s infectious illness	SMS	Every Sunday afternoon December and April	Parents
Teachers’ absence episodes and the number of days absent due to infectious illness	SMS	Every Sunday afternoon December to April	Teachers
School toilet facilities	Observations and internet-based questionnaire	One day between January and April	Hi Five researchers and children
Cleaning standard of school toilets	Bacteria samples, observations and internet-based questionnaire	One day between January and April	Tryptic Soy Agar (TSA) contact plates with Neutralize, Hi Five researchers and children

### Process evaluation

The Hi Five process evaluation is based on multiple data sources including questionnaires and qualitative interviews with children, teachers and school coordinators at participating schools, as well as observations of toilet facilities. The concept of process evaluation presented by Linnan and Steckler [50] formed the theoretical basis for the construction of questions for interviews and questionnaires [50]. The purpose of the process evaluation was to evaluate the implementation of the intervention components, to identify contextual factors that influenced the implementation process, and to contribute to the interpretation of the outcome results of the Hi Five intervention. Thorough description of the process evaluation is beyond the scope of this paper.

### Health economic evaluation

Resources used for the intervention (production and implementation costs of intervention material, extra cleaning etc.) have been systematically registered. These data provide basis for health economic analysis of the Hi Five study. The cost-effectiveness of the multifaceted implementation strategy will be evaluated from a societal perspective.

### Study status

The study is ongoing and the investigators are at present analyzing data.

### Did the randomization into intervention and control schools result in comparable groups?

#### Analyses strategy for test of randomization

Questionnaire baseline data were imported into SAS version 9.2. Self-reported baseline measures of gender, grade, family composition, number of siblings, illness-related absenteeism, hand washing behavior with soap, hand washing behavior after toilet use, hand washing behavior before eating lunch at school, use of school toilets and school satisfaction were included to characterize the children and assess the success of randomization. Ethnic status and family socio economic position (SEP) (I-VI) were included for school children in 5-8^th^ grade. Variables on ethnic status were categorized into ethnic Danish, immigrants, and descendants of immigrants according to the definitions by Statistics Denmark. Family SEP was measured by children’s responses to three items on father’s and mother’s occupation. The research group coded the children’s information on parental occupation into six groups according to the Danish National Institute of Social Research: social class I (high) to V (low) and a group VI covering parents who were receiving transfer income. The applied categorization is almost similar to the Registrar General’s Social Class measure often used in studies from the United Kingdom. The children were categorized, according to the highest ranking parent, into three groups, family SEP I-II (high), III-IV (medium), and V-VI (low).

At baseline, 8,438 children were enrolled of which 7,525 (89.2%) responded to the questionnaire by answering at least the first question in the questionnaire (2,427 children from intervention I schools, 2,427 children from intervention II and 2,671 children from control schools). At baseline there were no differences between intervention schools and control schools for children in grade 0-4^th^ when considering gender composition, living with siblings, illness-related absenteeism in the past week, school happiness, use of soap or use of school toilets. A few more children at control schools lived with both parents compared to children from the other two groups. Fewer children from intervention group II report to wash their hands before lunch everyday compared to intervention group I. For children in 5-8^th^ grade there was no difference between intervention groups and control group when comparing gender composition, living with both parents, living with siblings, illness-related absenteeism in the past week, hand wash after toilet visit and use of school toilets. There seem to be fewer children from higher SEP families in intervention group II compared to the population included in the other two groups. More children in intervention group II have an ethnic Danish background than in the control group. Fewer children in intervention II seem to be happy about their school compared to the other two groups. Fewer children from intervention group I wash their hands before lunch every day (Table 3).

**Table 3:** **Baseline characteristics of children from intervention- and control schools: socio-demographics and outcome measures**

	**0-4** ^**th**^ **grade**			**5-8** ^**th**^ **grade**		
	**Control group**	**Intervention group I**	**Intervention group II**	**Control group**	**Intervention group I**	**Intervention group II**
**Background factors**						
Number of schools	15	14	14	15	14	14
Number of school classes with responding children/invited number of school classes	74/75	68/70	70	59/60	55/56	53/54
Number of responding school children	1,525	1,392	1,430	1,146	1,035	997
**Socio-demographics**						
Boys (%)	50.4	49.2	49.9	49.7	47.6	49.9
Live with both parents (%)	75.7	72.3	72.1	66.8	65.7	64.3
Live with siblings (%)	91.2	92.0	91.8	90.2	89.0	89.7
Ethnic Danish (%)	-	-	-	87.6	89.5	91.1
Family socioeconomic position (SEP) (%)						
High (I and II)	-	-	-	41.8	45.1	38.9
Medium (III and IV)	-	-	-	42.8	40.6	48.1
Low (V and VII)	-	-	-	15.5	14.3	13.0
**Primary outcome**						
Illness-related absenteeism in the last week (%)	19.7	19.9	19.1	14.0	17.0	14.8
**Secondary outcomes**						
Use soap every time they wash hands (%)	83.7	83.4	80.6	60.8	62.3	56.0
Never/rarely forget to wash hands after toilet use (%)	64.2	62.7	63.5	83.8	85.0	82.1
Wash hands before lunch every day (%)	34.2	38.2	31.4	26.5	22.8	24.3
Never use the school toilet (%)	8.4	7.4	7.2	21.9	21.8	19.1
Happy about their school (%)	68.6	67.0	66.3	73.1	77.2	69.0

## Discussion

We have described the components of the Hi Five intervention and how it was developed. We have described how the design for the effect, process and health economic evaluation of the Hi Five intervention was planned. Further, we have found that overall the randomization into intervention and control schools resulted in comparable groups with only minor differences, mostly leaving intervention group II with a bit higher prevalence of adverse outcomes.

The novelty of the Hi Five study is that it is the first large-scale, school-based, multi-component intervention with a structural component (cleaning of school toilets) implemented for a whole school-year and evaluated with a long follow-up and in a randomized controlled design. To our knowledge, only one study has combined a structural intervention (e.g. a school disinfection program) with a hand hygiene component. This study was performed on a single school in Ohio. Intervention classrooms received alcohol-based hand sanitizer for use at school and quaternary ammonium wipes to disinfect classroom surfaces daily. The absenteeism rate for gastrointestinal illness was significantly lower in the intervention-group compared with the control group [25]. This study did not involve supplementary cleaning of the school toilets as an intervention component. The Hi Five study may contribute with new understanding and knowledge about the importance of school toilet facilities and optimizing of toilet facilities for children’s infectious illnesses and well-being.

Several hand hygiene interventions in school settings where educational efforts have been supplied with extra hand washing or use of hand disinfection have succeeded in reducing the number of infectious illnesses among school children [16-27], although one large randomized study showed no effect [3]. Internationally, we are aware of two large cluster-randomized studies in a developed country setting that have targeted school children at all grade levels, comparable to the Hi Five design. In New Zealand, a cluster randomized trial estimated the effectiveness of hand sanitizer in reducing illness absence episodes in children involving 68 primary schools. The study was not able to demonstrate an effect of hand sanitizer on illness-related absenteeism (3). The New Zealand intervention differed from that in Hi Five by: 1) allocating less time for the curriculum component, 1 lesson of 30 minutes versus 5–6 lessons of 45 minutes in the Hi Five intervention and 2) by introducing hand sanitizers instead of extra hand wash during the school day. In England, a large randomized controlled trial ‘Hands up for Max!’ including 178 schools examined whether an educational package to promote hand washing was effective in reducing absenteeism among children and staff in primary schools (21). Participating schools were randomized to receive the intervention in 2009 or to receive the intervention delayed after all follow-up data were collected in 2011 (control schools). The ‘Hands up for Max!’ educational resource pack used in the trial included a CD-ROM or DVD animation teaching how to wash hands correctly, lesson plans exploring ‘What are germs?’ and ‘Healthy hands, healthy school’, A4 posters demonstrating how to wash hands correctly, and stickers for children. The effect evaluation of this study is not yet published. The materials used in ‘Hands up for Max!’ are in many ways equal to that used in Hi Five. Neither the New Zealand study, the ‘Hands up for Max’ study or any of the previous smaller intervention studies have included education related to the importance of regular toilet use and good toilet manners as part of the study program.

Intervention studies have shown that hand sanitizers are an effective alternative to conventional hand washing [23,37]. Studies with a hand wash intervention have reported practical barriers: It takes up time, water spillage, and there are often an insufficient number of wash basin per child [14,37]. Although it would have been easier and less time consuming to implement hand sanitizers, hand sanitizers are only efficient when the hands are not visibly dirty, but hands of children in the youngest age groups are often visibly dirty. Secondly, we think it is important and a more sustainable intervention to teach children to wash their hands properly and to encourage regular hand washing. The Hi Five intervention may help habitualise this behavior at an early age. Thirdly, soap and water are the amenities available to children in most settings of their daily life, including their home. This is not the case for hand sanitizer.

The baseline data and the follow-up data were collected at the same period of the year, which is important to take into account the expected seasonal variation of the amount of infections. The study is aimed at all children at the school, because of the communicable nature of infectious illnesses. If only a selected number of school classes had been involved in the intervention, the effectiveness of the intervention towards reducing the amount of infections would have been be expected to be smaller because school children of all ages often share the same school rooms and toilet facilities. Although it was necessary to implement the intervention in all classes at all grade levels, our power calculation showed that we had enough statistical strength when only conducting the effect evaluation in one class at each grade level (0-8^th^). We did not include the 9^th^ grade, because the last year of schooling is focused on taking the finals and teachers and children often de-emphasize activities not relevant for finals. At schools with more than one class at each grade level, the classes were selected by random draw.

The use of new technological data collection methods, such as spoken questionnaires, SMS and MMS means that the project will also provide new knowledge about the applicability of these methods as means of data collection as a supplement to the information on children’s illness and illness-related absenteeism. To our knowledge, no former studies have collected data about children’s acute illness by SMS. The use of SMS, in order to obtain information about the children’s and teachers’ infectious illnesses and illness-related absenteeism makes it possible to repeat the data collection multiple times over a longer period of time and thereby reduce problems of recall. The Hi Five-study is a nationwide study involving public schools from all regions in Denmark. The schools were randomly chosen to be invited to participate in the study, and participation was optional. This may imply that the schools are not fully representative of Danish public schools. Schools with many resources or special interest in the area may have been more inclined to register for the study, or schools which have big concerns about their toilet facilities may have registered to a larger degree, because of their need of help to advocate for better facilities. After registering for the study, schools were randomly allocated to either intervention or control, a procedure which balances confounders in the assignment of treatment.

The Hi Five intervention was designed to be sustainable when the research team withdraws after the evaluation study, and non-participating schools should be able to take ownership of the Hi Five intervention and implement it without external help. We deviated from this aim, when we found finance for the extra cleaning instead of letting municipalities cover this extra expense. However, the intervention may be implemented without this part.

## Conclusions

The Hi Five study is a large, theory based, multicomponent school-based randomized controlled trial. It aims to reduce the prevalence of infections by 20% among children at intervention schools compared to control schools, and to improve hand hygiene and well-being. The intervention includes three main components: 1) a curriculum component 2) mandatory daily hand washing before lunch 3) extra cleaning of school toilets during the school day. The Hi Five intervention is evaluated by a large, randomized trial with systematic measurements and performance of process-, effect-, and health economic evaluations of the study. We collected quantitative and qualitative data from school children, parents, teachers and school toilets at baseline and follow-up. There was baseline equivalence between intervention- and control groups for sex, illness-related absenteeism in the past week and hands wash behavior after toilet visit. The Hi Five study fills a gap in international research. The study is the first to include education on healthy and appropriate toilet behavior as part of the curriculum and no previous studies has involved supplementary cleaning at the school toilets as an intervention component.

### Ethical issues

The Hi Five Study adheres to all Danish ethical standards and has been approved by the Danish Data Protection Agency, 1 October 2011, ref: 2011-54-1240 and reported to the regional ethics committee for the Capital Region of Denmark. They concluded that formal ethics approval was not required because no human biological material was sampled. There is no formal institution for ethical assessment and approval of questionnaire-based population studies in Denmark. When schools were invited to participate, written information was send to the school targeting the school leader, the teachers and parent boards and pupil councils at all schools explaining the implications of participation in the study. Teachers, children and their parents at the participating schools were informed that participation was voluntary, that their information would be used for research purposes only and treated confidentially and of the possibility of withdrawing during any stage of the study. Parents were informed of the study and the possibility to withdraw their child from the study by means of 1) a written form which was brought home from school by the child indicating the purpose of the study, the implication for and involvement of their child 2) by written information on the parental e- platform which is daily used by parents and school as means of communication.
